# Supplementary material for: Are carbon emissions trading and green financial instruments synergistic? -Comprehensive quantitative research based on content analysis
Source: PLoS One. 2024 Mar 7;19(3):e0298601. doi: 10.1371/journal.pone.0298601 (PMC10919601; doi:10.1371/journal.pone.0298601)
Supplement: S1 Data — (ZIP) [file pone.0298601.s001.zip › Data/地级市Z_F工作报告.zip!/║■─╧/╘└╤⌠╩╨/└┤╫╘╣1⁄2╓┌║┼í╛┬φ┐╦╩2╛▌═°í┐_2009.docx]

各位代表：

现在，我代表市人民政府，向大会作政府工作报告，请予审议，并请市政协各位委员和其他列席人员提出意见。

2008年工作回顾：在迎战困难中保持又好又快发展

刚刚过去的2008年，是很不寻常、很不平凡的一年。全市上下在中共岳阳市委的坚强领导下，深入贯彻落实科学发展观，大力推进“一化三基”战略，努力践行民本岳阳执政和发展理念，积极应对年初冰雪灾害、原油价格大起大落和国际金融危机等严峻挑战，完成了市六届人大一次会议确定的年度各项目标任务，实现了本届政府工作的良好开局。

—— 经济总量跨上千亿元台阶。预计全年实现生产总值1140.3亿元，比上年增长14％，其中一、二、三产业增加值分别增长4％、17.5％和13.9％，经济总量稳居全省第二位。全市财政总收入71.1亿元，其中一般预算收入42.9亿元，分别增长 17.6％和23.9％。县区经济发展各具特色，华容、湘阴、汨罗生产总值过百亿元，汨罗、岳阳楼区、岳阳经济开发区财政总收入过5亿元。

—— 新型工业化主导经济增长。全市新增规模工业企业124家，增加值增长20.2％；高新技术企业新增12家，增加值增长38％；私营企业新增1259家，非公有制经济占GDP的比重提高到53.1％；新增中国驰名商标3件、省著名商标28件。全市三次产业结构为15.8:51.2:33，二产业比重首次达到50％以上，对经济增长的贡献率达到61.6％。

—— 现代农业和新农村建设加快推进。粮食总产创历史新高，达到317.5万吨，增长10.8％；市级以上龙头企业180家，全市农产品加工业增加值增长16.2％；涌现了一批“村民富、村庄美、村风好”的新农村建设典型。

—— 新型城市化取得新进展。全市城市化率46.6％，提高1.5个百分点。我市成功通过国家卫生城市复查，继续被评为全国文明城市创建工作先进城市。更加注重生态环境建设，万元GDP能耗下降5%，主要污染物排放总量减少2.7％。

—— 三大需求保持较快增长。全市实现社会消费品零售总额358.3亿元，增长23％；接待境内外旅游者1150万人次，旅游总收入增长16％。全社会固定资产投资430亿元，增长34.5％。进出口总额8.5亿美元，增长14.1％。

—— 城乡居民收入不断提高。全市城镇居民人均可支配收入15344元，农民人均纯收入4750元，分别增长14.8％和10％。

—— 开放形象进一步提升。成功举办奥运火炬传递、端午旅游文化节、平江起义80周年纪念活动、第二届湘商大会、首届文化艺术节等重大活动。岳阳被国家商务部定为“加工贸易梯度转移重点承接地”，城陵矶港被定为海峡两岸首批货运直航港口，打造了新的开放平台。

我市经济社会发展取得的新成绩，进一步坚定了全市人民继续前进的信心和勇气。一年来，我们主要从五个方面开展工作：

一、科学谋划，争创发展空间

抓住国家批准长株潭为“两型社会”综合改革试验区的机遇，成功争取湘阴、汨罗、屈原部分地区及临港产业新区共875平方公里范围纳入核心区规划。在长株潭“两型社会”五大示范区中，岳阳争取了一个示范区——滨湖示范区。确定了长沙芙蓉北路延伸至湘阴、省道201南延至长沙、省道306改造等项目。完成了23.6平方公里的临港产业新区规划编制，启动了进港路、通港路等工程建设。抓住国家高度重视生态环境建设的机遇，加快洞庭湖综合治理规划和项目研究。基本完成《洞庭湖生态保护、环境治理与资源利用整体规划纲要》，建立洞庭湖综合治理项目库。“平安洞庭”项目通过国家评审，层山安全圈围堤加固和移民迁建工程开工建设，湘江、资江、汨罗江尾闾疏浚立项，华容河治理和城西、君山、屈原、麻塘等8个堤垸治理项目进入国家专项计划并开始启动。抓住国家土地政策调整的机遇，完善土地利用总体规划和城市总体规划修编。《岳阳市土地利用总体规划大纲》在全省最早通过审批，扩大市区和各县城规模控制面积，设定弹性区，预留了城镇发展空间。抓住国家扩大内需、刺激消费的机遇，加强服务业发展规划研究。编制《岳阳市旅游资源整合实施方案》并启动实施，完成航运物流业、城区服务业等发展规划编制，促进产业优化布局、集聚发展。

二、项目带动，支撑发展提速

2008年作为项目建设和项目服务年，全市上下形成了抓招商、争项目、促发展的浓厚氛围。精心组织“沪洽周”、“中博会”、广州农业产业化招商、东莞电子信息产业招商、精细化工产业投资推介等活动，扎实开展小分队招商。全市实际利用外资1亿美元，利用内资130亿元，分别增长25.5％和19％。项目建设加快推进，投资过亿元的新开工产业项目72个、在建基础设施项目102个。新型工业化项目：总投资165亿元的炼化一体化、总投资43亿元的华能三期、总投资25.7亿元的岳纸40万吨超级压光纸项目进展加快。赛隆制药、湖湘木业、鑫茂铜业、兆邦陶瓷、钰林服饰、拓邦电子等项目建成投产，海螺水泥、华润啤酒、国泰食品、桑乐太阳能、润华单晶硅、催化剂新基地、凯迪生物质发电等项目开工建设，旭宁软件、新瑞玻璃颜料、美国空气化工等一批项目落户岳阳。平江、云溪、荣家湾220千伏变电站相继开工。继续开展工业园区满园扩园行动，岳阳经济开发区和各县（市）区工业园产业特色更加明显。新型城市化项目：岳阳文化艺术会展中心投入使用，武广铁路客运岳阳站、洞庭风光带三期开工建设。青年中路、金鹗西路、南湖大道二期等城区主干道新建改造顺利完成。改造中心城区主干道人行道22条、小街巷63条，垃圾无害化处理、环卫设施配套等市政工程加快推进。实施公交优先，更新公交车231辆。城镇扩容提质步伐加快，平江、湘阴县城和张谷英、三封寺镇等面貌有新的改观。新农村建设项目：完成通乡通村公路2571公里，村级道路硬化率达到65％。建设农村客运站场12个，新建农家店670家，完成120个村配电网改造。新建农村沼气池2.4万口，改水、改厨、改厕7.5万户，新解决16.5万农村人口饮水安全。分批组织村干部外出学习考察，发挥农民在新农村建设中的主体作用。新型服务业项目：洞庭湖国际公馆启动建设，中坤、中融、新华雅等一批企业集团进驻岳阳投资大型旅游休闲项目；左岸巴陵、九天商厦等商业项目建成营运；天伦城、湘阴东湖等房地产项目有序发展。重点交通项目：城陵矶（松阳湖）港区一期工程及口岸联检设施、漕溪港码头一期工程竣工。随岳、岳常、岳潭3条高速公路和省道308湘阴段平江段、临鸭公路、汨罗江大桥、柳林江大桥等一批重点项目开工建设，国道106平江段、省道202华容段、铁桃公路、岳汨公路、岳荣新二期等改造进展顺利。

三、改革突破，化解发展难题

深化国有企业改制。进一步完善政策，着力解决资金筹措、资产盘活、职工安置等难题，完成28家市属国有企业改制，5600多名职工移交社区。各县（市）区共完成国有企业改制93家，安置职工11830名。启动集体林权制度改革。全面清理862万亩集体林地权属，在平江金塘村林改试点的基础上，全面铺开全市林改工作。推进农村土地有序流转。在新墙镇、黄市乡、南山乡、黄盖镇、羊楼司镇等地建立土地承包经营权流转服务中心，全市共流转土地45万亩，50亩以上的种养大户达到420户。开展城乡建设用地增减挂钩试点。湘阴、华容、临湘、君山试行增减挂钩、等量置换，新增城镇建设用地300公顷。实施城区新的财税体制。实行“同城同制、分税分成、属地征管、双保双赢”，市区两级事权与财权进一步统一。市本级财力支持县乡发展的额度加大，比上年增长33.5％。完善投融资体制。市国资公司规范运作，市中小企业信贷担保公司成立运营，组建临港产业新区开发建设投资公司，与市城建投、市土地储备中心构成多元投融资体系；汨罗等地融资成效明显。对20家企业开展上市辅导，凯美特上市进入中国证监会审核程序。加强银企对接，年末全市金融机构贷款余额比年初净增40亿元，商业保险规范发展，农业政策性保险全面推进，对地方经济支持作用加大。

四、环境整治，改善发展条件

治理环境污染。湘江、洞庭湖水系治理加快实施，57个综合整治项目相继开工，65家洞庭湖区造纸企业整治到位，37家非洞庭湖区造纸企业关闭23家、治理14家。南湖治污力度加大，蓝藻暴发得到较好控制，局部水域水质优于四类。大力推动城镇治污“三年行动计划”，完成蛇皮套、金东门排水整治，南津港二期、君山等8个污水处理厂启动建设，丰利纸业碱回收系统等12处重点治污项目顺利实施。城市环境综合整治定量考核继续居全省第二位。治理市容市貌。市、区两级整体联动，67个集贸市场、500多家“五小”门店和城乡结合部、城区卫生死角得到有效整治。市区新建高标准公厕16座、环卫垃圾屋343座，新增亮化景点140处，城区交通秩序逐步规范。通过创卫复查，广大市民对城市的责任感、认同感增强。整顿经济秩序。打击恶意逃废债务行为，开展金融诉讼案件集中执行活动，执行到位资金2.9亿元。我市被中国金融研究院、中国金融网评为“中国金融生态城市”。查处假冒伪劣食品药品案件857起，有效防止“问题奶粉”流入市场。加大打击传销力度，捣毁窝点612个。规范房地产开发，中心城区拆除违法建筑267栋。治理社会秩序。畅通信访渠道，开展大接访，有效解决了一批热点难点和历史遗留问题。深化安全生产专项整治，解决重大安全隐患18处。严厉打击各类违法犯罪行为，防控体系建设进一步加强，社会大局和谐稳定，社会治安综合治理工作居全省第二位。优化政务环境。认真贯彻《湖南省行政程序规定》，取消行政性收费62项，废止和宣布失效的规范性文件109件。完善政府绩效管理，开展创建服务型机关活动，实行重大投资项目行政审批委托代办，市、县政务服务中心运作更加规范高效。全面开展规范行政处罚自由裁量权工作，及时解决影响经济环境的突出问题，处理投诉210起，优化发展环境力度加大。

五、民生为本，共享发展成果

战胜历史罕见的冰雪灾害。全市上下众志成城，组织开展大救援、大分流、大除冰，保交通、保电力、保供应、保民生，没有因灾冻死、饿死一人。灾后迅速组织万名干部下基层，妥善安排群众生活，重建家园，把灾害损失降到最低限度。促进社会事业协调发展。免费义务教育覆盖城乡，新建合格学校85所，湖南民族职业学院通过教育部高职高专人才培养工作水平评估，出台了支持民办教育发展的政策。推进产学研结合创新，各类工程技术中心达到16家，具有自主知识产权的高新技术产品348项。新型农村合作医疗覆盖面达到90.5％，新建改造乡镇卫生院40所；建设城市社区卫生服务机构65家，形成“15分钟医疗卫生服务圈”。人口计生工作在全省保先进位，华容、汨罗分别保持和进入“全国计划生育优质服务单位”。举办全市第二届大众运动会。落实文化惠民，公共文化建设得到加强，网络的影响力日趋广泛。全面完成省市实事项目。我市承办的省实事28项任务34项指标、市10件实事29项指标全面完成。新增城镇就业5.7万人，新增农村劳动力转移就业9.8万人，城镇零就业家庭动态清零873户；养老、医疗、失业、工伤、生育保险征缴社保基金20亿元，三年翻一番；城镇居民基本医疗保险全面启动，参保68.6万人；新建改造乡镇敬老院55所，在全省率先实现“乡乡有敬老院”；新建农村安居房2202户、城镇廉租房1140户、经济适用房16万平方米，1.28万户居民享受廉租房租赁补贴。认真落实各项惠民政策。各类支农补贴及时发放。城乡低保人均月补差分别达到100元和36元。企业军转干部和军队退役人员生活补助进一步提标。取消工商“两费”。深入开展农业技术下乡活动，推进便利消费、便民服务进社区、进家庭，为群众带来更多实惠。

一年来，全市上下深入学习实践科学发展观，开展解放思想大讨论，加强精神文明建设，为经济社会发展提供了强劲动力。切实加强民主法制建设和政府自身建设，推进科学民主决策。共承办人大议案、代表建议309件，承办政协建议案、委员提案508件，办理行政复议案22件，人民群众满意度提高。村民自治巩固发展，社区建设取得好的成效。坚持“不换状态就换人”，公务员队伍建设加强。深入开展反腐败斗争，查处违法违纪案件755件。国防教育、国防动员和后备力量建设不断加强。审计、统计、质监、气象、史志、档案、保密、盐业、烟草、老龄、对台、民族宗教、外事侨务、新闻出版、广播电视、社会科学、邮电通信等工作都有新的进步。

各位代表！过去的一年，在诸多困难制约下，能够取得这样的成绩，确实来之不易。这得益于市委总揽全局、超前谋划、强力推进，得益于市人大依法监督、市政协民主监督和大力支持，得益于改革开放30年奠定的良好基础，凝聚着全市人民的辛勤劳动和不懈努力。在此，我代表市人民政府，向全市人民，向所有关心、支持和参与岳阳改革建设的人们，致以崇高的敬意和衷心的感谢！

我们也清醒地看到，全市经济社会发展还面临不少困难和问题。主要是：一般预算收入增幅放缓，财政收支压力增大，难以满足公共财政的需求；经济结构调整步伐不快，一产业比重仍然偏大，三产业还没有形成特色，高能耗、高污染企业比重偏高；农业基础薄弱，抗灾避灾机制不健全，农民持续增收乏力；国企改制任务艰巨，体制矛盾比较突出；就业、环保、安全生产等压力加大，维护稳定的任务还很艰巨；社会管理和公共服务相对薄弱，人民群众还有很多不满意的地方；发展环-境有待进一步优化，还存在执法不公、政令不畅、效率不高等问题和消极腐败现象。对此，我们一定高度重视，积极采取措施，认真加以解决。

2009年目标任务：攻坚克难、科学跨越，保增长、保民生

2009年，是我市经济社会发展极为关键的一年。我们面临前所未有的严峻挑战：一是全球金融危机引发实体经济衰退，带来市场低迷，对我市纺织、化工、能源等基础产业影响很大；二是国家为刺激经济增长实施减税让利政策与经济增速下滑的双重挤压，将使我市可用财力下降，保运转、保民生任务艰巨；三是返乡农民工增多与经济增速放缓，将进一步增加就业、社保和稳定压力；四是节能减排、保护环境任务更加艰巨，经济社会发展制约因素增多。我们也面临前所未有的发展机遇：一是国家实施积极的财政政策和适度宽松的货币政策，正是我们上项目、争投资、加快基础设施建设的好时机；二是国家实施扩大内需、刺激消费、改善民生的一系列政策措施，正是加快发展旅游业和房地产业、拉长三产业短腿的好时机；三是国家增值税转型和建设成本大幅降低，正是鼓励企业加快技术改造、提质升级、积蓄发展后劲的好时机；四是随着沿海部分产业向中西部转移，正是我们扩大招商引资、优化经济结构的好时机；五是我市部分地区纳入长株潭“两型社会”滨湖示范区，正是我们加快建设资源节约型、环境友好型社会的好时机；六是经过改革开放30年特别是近几年来的发展，我市经济基础得到有力夯实，后劲增强、态势良好，全面提速、加快发展的基本面没有改变。我们一定要提振信心，特别是提高企业的投资信心，提高群众的消费信心，提高干部的工作信心，凝聚全市上下的智慧和力量，共克时艰，促进经济社会又好又快发展。

今年政府工作的总体思路是：深入贯彻党的十七大和十七届三中全会精神，以科学发展观统领经济社会发展全局，大力推进“一化三基”战略，积极践行民本岳阳执政和发展理念，强力实施民本岳阳九项工程，攻坚克难，科学跨越，突出扩大投资、扩大消费、保经济增长、保民生改善，加快实现“产业兴旺、城乡秀美、文化繁荣、民生殷实、社会和谐、走势强劲”的富民强市目标。

今年全市经济社会发展的主要预期目标是：全市生产总值增长11％；财政总收入增长12％；全社会固定资产投资增长30％；社会消费品零售总额增长16％；城镇居民人均可支配收入和农民人均纯收入分别增长12.4％和11％；城镇登记失业率控制在4.5％以内；万元GDP能耗下降4％，主要污染物排放总量减少2.7％；森林覆盖率36.1％；人口自然增长率控制在6.2‰以内，各项社会事业协调发展。

一、突出基础设施建设

坚持把扩大投资作为保增长、促发展的核心措施，抢抓国家政策机遇，打破常规抓项目、争项目、上项目。

加快交通项目建设。对外打通进出通道，对内加快成网成环，提升综合交通枢纽地位。高速公路：加快推进随岳、岳常、岳潭高速公路建设，启动岳汝高速公路建设，加快与长常、京珠高速合龙成环，建设对外高速通道。干线公路：加快在建的“十路两桥”项目建设。启动芙蓉大道湘阴段和岳汨公路延伸段建设，加快省道207平江段、省道202二期、省道306岳阳至平江段、鹿白公路、梅成公路升级改造，完成京珠高速羊楼司连接线建设，启动鲇鱼须大桥建设，争取湘阴湘江二桥尽快立项。改造建设农村公路2000公里，新建农村客运站场12个。做好政府投资建设的二级公路收费站撤除的相关工作。铁路：继续支持武广铁路客运专线建设，重点抓好岳阳站、汨罗站及配套工程建设；开工建设荆岳铁路；开展岳九、岳常铁路项目前期工作。水运：启动岳阳至武汉长江航道疏浚和炸礁工程，争取航道维护尺度最低标准由3.2米逐步提高到4.5米，5000吨货轮可以常年通航；加快漕溪港二期、营田、鹿角、鸭栏、塔市驿、荆江门等码头项目建设，开工建设洞庭湖水上救援中心和长航岳阳综合码头。航空：做好岳阳机场前期工作，力争列入国家“十二五”建设计划。

加快农业基础设施和新农村建设。推进流域治理。继续加强洞庭湖综合治理，加快层山安全圈建设。抓好湘江、华容河等水系治理，力争汨罗江、新墙河等中小河流清淤治理项目进入国家规划。推进农田水利建设。抓好铁山灌区续建配套、团湾水库渠系配套工程建设，抓好燎原、北汊等中型水库及一批重点小一型病险水库除险加固；实施麻塘、钱粮湖、隆西、幸福、屈原等堤防加固工程；加快洞庭湖排涝设施建设，推进花兰窖、悦来河、谷花洲、磊石、城西等11座大型排涝泵站更新改造。建设预警预报系统，增强农业防灾抗灾能力。推进农村生态环境建设。加快沼气池建设，加快东洞庭湖湿地农业综合利用示范区、巩固退耕还林成果配套项目建设，完成6.5万亩林业血防工程建设，启动岳阳县30个自然村清洁示范工程试点。推进农村公益设施建设。加快推进平江、华容等县城引水工程，新解决20万农村人口饮水安全；继续实施广播电视“村村通”工程、电话“村通工程”和信息“金农工程”。以基础设施的完善提升新农村建设水平，坚持规划先行、点面结合、政策引导，激发广大农民和全社会参与新农村建设的热情。

加快城市基础设施建设。围绕建成更加适宜人居、旅游、创业创新的沿江港口城市和特色显、品位高、实力强的湘北门户城市定位，加快新型城市化进程。扩城：东扩武广铁路客运岳阳站新片区，完成站场建设，延伸巴陵东路，启动建设金凤桥路和皇姑塘立交桥，加快新区开发；南延湖滨休闲旅游区，完成南津港大堤拓宽改造，启动园艺路建设，吸纳更多项目进区；北靠城陵矶临港产业新区，改造延伸望岳路和冷水铺路，促进中心城区与港区全面对接；西连君山生态区，以岳常高速、荆岳铁路建设为契机，推进跨湖发展。提质：加快洞庭风光带三期、巴陵广场、环南湖旅游走廊建设。完成枫桥湖路、青年中路、建湘路延伸改造；抓好洞庭大道、桐子岭路、长寿巷、景湖路等城市主次干道改造。启动实施一批旧城改造项目。中心城区新建一批公厕、垃圾屋，改造43条小街巷。绿化：启动珍珠山公园建设，抓好金鹗公园南大门广场和长虹路、东环路等绿化改造。机关、企业和社区都按照绿化规划的要求，见缝插绿、拆围透绿、异地补绿。治污：整体策划南湖、东风湖、芭蕉湖、王家河等水系综合整治，加快项目论证和规划编制，分步实施，逐步改善城市水系与水质。推进环南湖截污管网建设，完成南津港二期等8个污水处理厂和县（市）区10个无公害垃圾处理场建设。把县级市、县城和中心镇作为推进新型城市化的主要节点，扶持一批基础条件好的建制镇发展为经济强镇和特色小镇，加快形成市区引领区域发展、县城支撑县域经济、小城镇聚集经济要素的新型城镇体系。

加快临港产业新区及滨湖示范区建设。临港产业新区建设，按照“产业高地、试验新区、临港新城”的定位，强力推进基础设施。尽快建成进港路、通港路，整合新老港区资源，启动云溪化工专用码头、货运铁路专线建设，抓好管网、通讯、供排水、供电、供气等配套建设。充分利用口岸优势和岸线资源，引进战略投资者临港布局大项目，促进产业分区聚集扩张。推进大通关建设，尽快启动台海首航，加强与上海、武汉等地通关协作，实现跨关区无障碍通关。滨湖示范区建设，重点抓好空间布局、产业发展、土地利用和基础设施规划编制，加快连接长株潭进出通道建设，以制造业、现代农业和物流业为重点加快产业对接。

二、突出重大产业发展

按照产业集群、园区集聚、发展集约的思路，抓好重大产业发展，大力推进新型工业化，带动现代农业、现代服务业加快发展。

抓好推进新型工业化的重大产业。把新型工业化作为经济发展的第一推动力，力争全年新增工业投资150亿元，规模工业企业达到1200家以上，增加值增长13.5％。

加快重大产业项目建设。石化产业：围绕炼化一体化、催化剂新基地等大项目，加大服务力度，优化建设环境。加快云溪精细化工园及长炼分园、巴陵石化分园和临湘儒溪农药化工园建设，引进一批大型化工项目落户，重点抓好大厂产品深加工和配套产业发展，进一步拉长产业链、扩张规模、提升产能。造纸产业：支持岳纸40万吨超级压光纸项目建成投产，争取新上50万吨高档文化纸项目，鼓励丰利、丰隆纸业等提升水平。能源材料产业：加快华能三期建设，争取新上君山、黄盖湖风力发电和平江抽水蓄能发电项目。继续做好华容小墨山核电、湖南电煤储备基地等项目前期工作，力争进入国家“十二五”规划。加快新一轮大规模电网建设改造，完成3个在建的220千伏变电站建设，投运古塘、公田、松阳湖、鹿角110千伏变电站。争取海螺水泥、兰峰水泥粉磨站等项目尽快建成投产。机械制造产业：支持特种电机、汽车空调、农用机械、潜泵、电磁铁等优势品牌改造扩张，支持在岳军工企业发展民品生产，支持创普船舶重工、伟业造船、岭北船舶等制造项目加快建设。生物医药产业：支持科伦药业、同联药业、景达生物等加快发展，规划建设生物医药产业园，推动医药物流等关联项目建设，打造全国重要的抗生素和大输液生产基地。新兴产业：加快桑乐太阳能、润华单晶硅、龙华电子、旭宁软件等项目和田谷电子信息产业园建设。加强信息基础设施建设，加快邮电通信业发展。支持汨罗循环经济发展，支持泰格林纸启动全国循环经济试点。

扶持企业解困发展。切实把中央扶持企业发展的一系列政策措施细化具体、落实到位，使企业稳得住、发展好、有后劲。鼓励银行在风险可控前提下，加大对企业的信贷支持力度，完善中小企业信用担保体系；落实税收优惠、缓缴社会保险费、降低社会保障费率、扩大失业保险基金支出范围等政策措施；支持发展一大批中小企业，与大厂对接、向园区聚集，增强产业配套能力。进一步加快工业园区建设，争取岳阳经济开发区进入国家级开发区行列，各县（市）区工业园形成特色鲜明、竞相发展的县域经济增长极。

增强企业自主创新能力。支持企业加快技术改造，加强产学研结合创新，开发具有自主知识产权的高新技术装备及产品，重点突破优势产业关键共性技术和瓶颈技术，推动产品优化升级。实施品牌战略，推进国家知识产权试点城市建设，加大自主创新引导力度。争取国家电磁铁质量技术检测中心落户岳阳。力争全市新增高新技术企业20家以上，各类技术工程中心达到20家，工矿企业专利授权量增长20％，增创一批驰名商标和著名商标。

抓好推进农业现代化的重大产业。全面贯彻落实十七届三中全会精神，以新型工业化理念引领农业现代化，提升农业综合生产能力，促进农民持续增收。

发展特色大型基地。重点抓好大型商品粮基地、5亿斤粮食产能基地、优质粮食产业工程和市、县两级粮食储备项目建设，力争全市粮食生产面积突破800万亩，粮食产量超过335万吨。抓好100万亩优质油茶、生猪健康养殖、名特优水产养殖、无公害蔬菜生产和竹木林纸等基地建设。加强耕地质量建设，推广粮食、油菜、棉花等大宗农产品品种改良。着力形成一批区域优势突出和特色鲜明的产业带，放手发展大户经济，提升规模型、标准型农业水平。

做强龙头加工企业。加快君山全国农产品加工示范基地建设。重点培育壮大旺云米业、盈田米业等粮食加工企业，巴陵油脂、义丰祥、鲁良新元等食用油加工企业，李记食品、十三村、插旗菜业等蔬菜加工企业，君山银针、兰岭、洞庭春等茶叶加工企业，芭蕉扇业、羊楼司竹业等竹木加工企业；支持华润啤酒、岳阳楼啤酒、胜景山河黄酒等酒类企业，加华牛业、正虹海原等肉类加工企业加快发展。加强地理标志证名商标、集体商标等特色农产品品牌建设，争取水产品精深加工有新的突破，联手打响“洞庭湖”品牌。通过农产品加工龙头企业连基地、带农户，构建引领产业化的带动力量，提升加工型、品牌型农业水平。

健全农村服务体系。围绕种养、加工、销售等经营环节，种苗、技术、防疫等服务环节，加强政策支持、金融服务、法律服务、信息咨询，发展多种形式的农民专业合作组织。提高农业机械化装备水平，推动农业科技创新。加大对农产品和农资生产经营的监管力度，严厉打击坑农害农行为。

抓好推进现代服务业的重大产业。着力提振消费信心，引导消费预期，改善消费环境，培育消费热点。

加快房地产业发展。把房地产业作为城市经济的支柱产业，采取特殊措施推动加快发展：一是规范房地产市场。严禁无资质开发，严禁行政企事业单位集资建房，严禁城市规划区内村民分散建房。二是优化开发结构。加快建设中低档、中小户型商品房，适当建设配套完善、品质优良的高档小区，满足不同层次的住房需求。三是改善开发环境。推动水、电、路、气等配套设施同步跟进，为项目开发和入住创造条件。四是扶持房地产企业。严格规范规费项目和标准；对实行土地招拍挂全额交缴出让价款的新项目，免收基础设施配套费；下调房地产开发项目资金监管比例20个百分点；推行房地产项目统一竣工验收。五是促进住房消费。在中心城区购买自住房，公积金贷款提高到30万元，还贷期限延长到25年；个人首次购买144平方米以下的普通住房契税率下调至1％。力争全年完成房地产投资35亿元以上。

加快旅游产业发展。全面落实《旅游资源整合实施方案》，推动岳阳旅游业转型升级和发展升温，逐步建成知名的旅游目的地。以“楼、岛、湖”整合为突破，对内统筹开发，对外抱团营销，与热点城市、精品线路、著名旅行商加强对接，做强旅游核心品牌。以“生态旅游年”为主题，支持洞庭湖国际公馆、中坤休闲度假基地、盘石洲生态园等项目加快建设，带动农家乐、水上游等休闲旅游全面提质。以旅游设施配套为基础，推进旅游景区沿线道路硬化、环境净化、生态绿化、农舍美化和旅游标识规范化，让游客有好的第一印象和整体印象。以特色地域文化为取向，运用市场手段办好端午龙舟节、洞庭湖观鸟节、湖鲜美食节，发掘中心城区圣安寺、乾明寺、吕仙道观等宗教资源，支持张谷英、屈子祠、龙窖山申报世界文化遗产，推动旅游与文化的融合。以提升服务水平为重点，新创5A景区、新上五星级酒店、新建岳阳游客服务中心，带动吃、住、行、游、购、娱全面升级，加快特色餐饮、土特产品、会展经济等发展，扩大旅游消费。

加快现代物流业发展。建设大市场集散物流。加强对周边市场的研究，进一步提升太阳桥、泰和等区域性市场，启动建设粮食物流交易中心、烟草进出口基地及配送中心，改建花板桥、岳城标准化集贸市场，形成一批带动加工、辐射周边的特色专业批发市场。依托大交通吞吐物流。发挥城陵矶港东进出海的作用，积极引导货源进港吞吐，做强航运物流业。借助岳阳铁路、公路和水运综合交通优势，推动物流向长三角延伸、向珠三角扩散、向西部推进。服务大产业拉动物流。大力发展第三方物流，支持各类中介业加快发展，提升生产性服务业水平。启动炼化公路建设，打通大厂物流瓶颈。构建中心商务区繁荣物流。大力发展现代商务，支持东茅岭路服装品牌、德胜南路湖鲜美食、巴陵东路汽车品牌、汴河创业街等特色商业街提升品位、做大规模，发挥沃尔玛、心连心、福润多等对物流的带动作用，吸纳地方产品入市。高度重视发展农村流通，继续实施“万村千乡工程”，推动“家电下乡”，以最终消费拉动中间消费，带动物流业发展。

三、突出民生改善

把保障和改善民生摆到更加突出的位置，让全市人民共享改革发展成果。

努力做好就业和再就业工作。把保就业作为保民生的头等大事，全面落实积极的就业政策。强化创业和就业培训，重点帮助下岗职工、失地农民和返乡农民工就业，继续巩固零就业家庭动态清零。促进农村劳动力转移，重视高校毕业生就业。实施全民创业工程，优化自主创业环境，以创业带就业。改进再就业服务中心工作，发挥其引导就业的积极作用。全年新增城镇就业4万人、下岗失业人员再就业2万人。发展和谐劳动关系，依法维护劳动者合法权益。

加快完善社会保障体系。继续扩大社会保险覆盖面，城镇职工五大社会保险共新增参保14.4万人以上，城镇居民基本医疗保险参保70万人以上。推进企业退休人员社会化管理服务，加快出台失地农民养老保险的具体办法。发挥商业保险在社会保障中的积极作用，规范保险市场，强化保险理赔。继续完善城乡社会救助体系，巩固城乡低保动态下的应保尽保，年内城乡低保人均月补差分别达到110元、40元。加大救济救助力度，积极发展社会福利、慈善捐赠和残疾人事业，加快市儿童福利院、市残疾人康复就业综合服务中心建设。

推动教育事业协调发展。强化政府对义务教育的保障作用，推动公共教育资源向农村、贫困地区倾斜。引导社会力量帮扶困难学生就读，关心留守儿童教育。加强教师队伍建设，认真落实义务教育阶段教师平均工资水平不低于当地公务员平均工资水平的政策。整合优化城乡教育资源，建设80所合格学校；抓好中心城区中小学校建设和教育布局调整，解决新扩城区学生就读难问题。支持湖南理工学院申报硕士点，支持岳阳职业技术学院申报省示范性高职院校。

扎实抓好卫生和人口计生工作。扩大公共卫生服务，加强预防保健和重点传染病防控，保障人民群众健康。加快市公共卫生应急指挥监督中心和市二医院科教大楼建设，改扩建17家城市社区卫生服务机构。启动县级人民医院标准化建设，每个县（市）区建设一家重点中心卫生院，实现一村一个卫生室。稳定新型农村合作医疗制度，参合农民的补助标准提高到每人80元。推广“服务零距离、药品零差价、医患零纠纷”社区卫生服务模式，降低就医费用。深化医药卫生体制改革，实施基本药物使用制度。坚持基本国策，狠抓计划生育基层基础工作，落实利益导向，加强农村和流动人口计划生育管理，治理出生人口性别比偏高问题，实现“保先进位创优”目标。

构建公共文化服务体系。大力实施精神文明建设工程，广泛开展文明社区、文明村镇、文明道路、文明单位等创建活动。加快发展文化事业和文化产业，加强广场、社区、校园、企业文化建设。启动市广播电视中心建设，新建一批社区和乡镇综合文化站、农家书屋，放映城乡公益电影3.5万场，广泛开展科技、卫生、文化“三下乡”活动，促进文化资源共享。做好青少年思想道德教育工作，加强网络平台建设和管理。加快市体育中心项目前期工作，启动市游泳馆建设，规划实施60个“农民体育健身工程”，有重点地发展竞技体育。

推进安居工程建设。加快建设保障性住房，改进实物配送，全面启动经济适用房货币补贴，新建经济适用房15万平方米，新建改建廉租房8万平方米。将市区38万平方米危房改造、城中村改造、铁路周边危房改造与保障性住房建设统筹推进。加快农村安居工程建设，新建农村安居房2000户、村级“五保之家”50个，改扩建乡镇敬老院20所。帮助洞庭湖区渔民逐步解决上岸定居、子女就学等问题。

全面落实各项惠民政策。认真落实各项支农惠农补贴政策，增加农民收入。加大扶贫工作力度，做好水库移民后扶工作。认真落实企业军转干部、退役士兵、伤残军人等解困政策，调整增加企业退休人员基本养老金。面向困难群众和弱势群体，继续办好一批实事项目。

四、突出改革开放

今年改革开放必须迈出更大步伐，为经济增长破解难题、化解制约、增添活力。

推进农村改革发展。按照加强农业基础、增加农民收入、保障农民权益、促进农村和谐的要求，加大农村改革力度。全面实施集体林权制度改革，保障农民经营自主权、处置权和收益权，逐步形成集体林业的良性发展机制。赋予农民更加充分而有保障的土地承包经营权，鼓励和引导流转，推动规模经营。深化户籍制度改革，逐步实行统一的户口登记管理制度。深化乡镇机构改革，在化解义务教育债务的基础上，努力化解乡村其它债务。健全政策性农业保险制度，扩大保险覆盖面和保险品种。推进基层水管单位体制改革，搞活农村小型水利设施经营权。

深化国有企业改革。完善国有资产监管体系，加强对国有企业资产转让、出租、出借、抵押及重大投资管理。充分发挥市国资公司融资平台作用，通过银行借贷融资、土地收储融资、上市公司融资和企业招商融资等形式，突破改制资金瓶颈制约。进一步完善职工安置、人员移交、证照办理、资产处置四个环节的具体实施办法，尽快完成国有企业改制任务。通过深化改革，支持非公有制经济扩张发展。

推进金融改革。支持各类金融机构改革发展，继续改善金融生态环境，推动银企合作，拓宽融资渠道。争取发行市政债券，支持市商业银行改革做强，力争年内新引进一家股份制商业银行、新建一家农村村镇银行、新增一家上市企业，推动金融与地方经济互促发展。

推进土地管理改革。扩大城乡建设用地增减挂钩试点范围，开展先征后转工作试点，保障民生项目和符合国家产业政策项目的用地需求。积极盘活城市规划区内长期闲置、低效利用的存量土地，对土地利用计划实行差别化管理。探索建立建设用地指标交易平台，改进报批方式，推进集体建设用地流转。建立耕地保护区域补偿机制，力争耕地占补有余。完善征地补偿制度，保障失地农民权益。

进一步扩大开放。敞开门户，在更广领域引进战略投资者。盯住世界500强企业、中央企业、大型民营企业和上市公司，依托优势资源、优良企业和优质项目，以高层接触、中介联系、网络发布、小分队出击等多种形式，提高招商引资实效。加强区域合作共赢，在巩固珠三角招商阵地的基础上，开辟长三角和台湾新领地，打造港澳、对台招商新平台，更好地承接产业转移。力争全年引进内资130亿元、外资1亿美元以上。敞开门户，让更多企业走出去做大做强。鼓励市内优势企业利用品牌开拓市场，利用外部资源建立生产和原料基地，利用人力和技术优势承包项目。推动加工贸易转型升级，扩大进出口，实现开放型经济的新突破。敞开门户，从更宽视野培养引进人才。树立人才是第一资源的观念，完善人才引进政策，重点引进高端人才，推进市场配置人才资源，营造优秀人才脱颖而出、人尽其才的良好氛围。

五、突出管理服务

新形势新任务迫切要求我们加强和改进管理。顺应人民群众的呼声，突出抓好社会安全、生态环境、城市管理等公共管理；针对行政运行的薄弱环节，突出抓好规划、投资、财政等政府内部管理。今年力争在这些重点领域和关键环节实现管理的规范化、精细化。

强化社会安全管理。进一步加强社会治安综合治理，集中整治突出治安问题，深入开展“扫黑除恶”、打击“两抢一盗”、整治地下“六合彩”、禁毒扫黄等专项斗争，增强人民群众安全感。全面落实安全生产责任制，切实加强生产安全和消防安全隐患排查整治，有效防止重特大安全事故发生。继续整顿规范市场经济秩序，实行食品药品管理体制改革，确保食品药品安全。依法打击制假售假、商业欺诈、非法集资和传销。加强信访和人民调解工作，健全利益协调、诉求表达机制，有效化解不安定因素。推动应急平台体系建设，提高突发事件的预防和处置能力。积极推进国防动员和后备力量建设，加强全民国防和国家安全教育，深入开展双拥共建。

强化生态环境管理。按照建设“两型社会”的要求，严格守住保护耕地、改善生态、节能减排、保障民生“四条底线”。推进节能减排，拒绝环保不达标项目进入，抓好金成实业、大地化工等30个重点节能项目，抓好巴陵石化污水处理、华能一期脱硫等16个重点减排治污项目。推进流域污染防治，对重点水系、重点区域、饮用水源地明确标准、落实措施、严格考核。推进农村面源污染治理，特别是畜禽、水产养殖污染治理，严控重污染企业和高耗产能向农村转移。推进土地、森林、矿产等资源保护和集约利用，严禁乱采滥伐。强化各级政府节约资源、保护环境的责任，增强全社会生态环保观念。

强化城市管理。巩固国家卫生城市创建成果，启动国家交通管理模范城市、环保模范城市创建活动，使城市更为洁净、更有秩序、更加生态。围绕加强市政、市容、交通和环卫等管理，建立长效机制。继续推进相对集中行政处罚权工作，创新城管综合执法方式。将管理重心下移，强化基层责任，把管理精细到路段、街巷，延伸到城中村、城乡结合部，实现环卫专业保洁和社区保洁的无缝对接。对市政维护、园林绿化、环卫保洁等引入竞争机制，加快市场化进程。开展城管志愿者行动，开展交通、卫生文明劝导，开展文明市民、社区、街道评比竞赛，切实解决夜市扰民、占道经营、乱停乱靠等问题，促进城市和谐。

强化规划管理。发挥规划对优化资源配置、实现战略目标的先导作用。把规划编制到位，年内完成市区70平方公里控详规划、岳阳市风貌规划和重点产业振兴规划编制，促进总体与分区、建设与土地、城市与农村、产业与生态等各类规划相互对接。把规划实施到位，强化规划的刚性约束，严格规划审批程序，严格规划监督程序，严查违反规划行为，对违规用地坚决纠正，对违法建筑坚决拆除。

强化投资管理。今年将是我市历史上投资最多的一年，管理不到位，不但不能促进经济社会发展，而且将带来财政收入减少、国家财产损失等一系列负面影响。对国家投资和政府融资的建设项目，进一步规范审批程序，严格实行招投标，严防滞留截留、挤占挪用项目资金，坚决杜绝“豆腐渣”工程、“烂尾”工程。对涉及公共安全的所有投资项目，切实加强质量、安全、功能等方面的监管。做到既增加投融资总量，又规避投资风险。

强化财政管理。应对国家实施积极的财政政策和增值税转型改革的新形势，采取有力措施破解短收难题。坚持依法征税，对漏征漏管的部门、行业和企业开展专项整治，加强增值税、所得税管理，确保应收尽收。对接基础设施、环境治理、结构调整、社会保障、支农直补等政策，争取国家更多财力支持。牢固树立过紧日子的思想，今年一般行政支出零增长、严格控制行政事业单位购置车辆、严格控制审批财政性投资的楼堂馆所。强化预算约束，把更多的财政资金投向民生领域、公益事业和困难群体。

六、突出政府自身建设

今年政府工作面临着前所未有的机遇、挑战和压力，政府工作人员必须有坚定的信心、创新的锐气、进取的状态，切实履行职责，努力建设人民满意政府。

推进依法行政，加强民主法制建设。认真执行人大及其常委会的决议决定，依法报告工作；尊重和发挥人民政协的职能，密切与各民主党派、工商联、无党派人士的联系；自觉接受人大法律监督和政协民主监督；认真办理人大议案、代表建议和政协建议案、委员提案。全面执行民族、宗教、侨务和对台政策，支持工会、共青团、妇联等群团组织依照法律和各自章程开展工作。扩大基层民主，完善政务、厂务、村务公开，推进村民自治，保障人民依法直接行使民主权利。加强普法教育和政府法制工作，提高政府工作人员法律意识，提高政府依法管理社会的能力。

转变政府职能，优化经济发展环境。把工作重点、政策着力点放到服务市场主体、优化发展环境上。突出加强项目服务，提升政务服务中心的功能，推进建设工程项目联合审批与联合验收、重大投资项目行政审批委托代办。特别是在争投资、上项目方面，打破常规，特事特办。突出规范行政行为，继续抓好规范行政处罚自由裁量权工作，严厉查处行政不作为、乱作为。严厉打击扰乱企业和工程建设环境的行为，严禁以各种名目向企业拉赞助、搞摊派，取消一切未经市委、市政府批准的检查评比。突出推进体制创新，继续深化政府机构改革、事业单位改革和行政审批制度改革，进一步减少行政审批，整合部门职能，形成保增长、促发展的强大合力。

强化制度建设，努力提高行政效能。认真实施行政程序规定，不断创新政府管理理念和方式手段，从行政运行的各个环节进行科学的制度安排，用制度管人管事。坚持按程序决策，进一步完善专家论证、公众参与、社会公示和新闻发布制度，推进决策民主化、科学化。坚持高效率办事，进一步优化办事、办文、办会流程，推进电子政务，加快政府门户网站、内网平台和城市综合管理平台建设，以信息高速推动政府工作提速。坚持凭绩效奖惩，进一步完善政府绩效管理，严格实行有错无为问责。

坚持执政为民，加强公务员队伍建设。牢固树立公仆意识，践行“两个忠诚”，为人民服务、对人民负责、受人民监督、让人民满意。大兴学习之风，深入持久地进行改革开放宣传教育，提高科学发展水平。大力弘扬艰苦奋斗和勤俭节约的风尚，坚决反对铺张浪费，推进节约型机关建设。坚持从严治政，把廉政建设与经济社会发展一同部署、落实、检查和考核，加强行政监察和审计监督，严肃查处各种腐败行为。按照“不换状态就换人”的要求，进一步完善公务员激励约束机制，关心爱护基层干部，激励政府工作人员在各自岗位上勇于创新，敢于突破，争创一流业绩。

各位代表！在大浪淘沙的市场经济中，在机遇挑战并存的前进道路上，我们有责任、有信心推动岳阳经济社会发展的新跨越。让我们高举中国特色社会主义伟大旗帜，以邓小平理论和“三个代表”重要思想为指导，深入贯彻落实科学发展观，在中共岳阳市委的领导下，进一步解放思想、抢抓机遇、团结拼博、开拓奋进，努力开创民本岳阳和谐崛起新局面，以优异成绩迎接新中国成立60周年！
